# Supplementary material for: COVID-19 impact data for the CHAMPS HDSS network: Data from Harar and Kersa, Ethiopia
Source: Data Brief. 2023 Aug 21;50:109508. doi: 10.1016/j.dib.2023.109508 (PMC10471914; doi:10.1016/j.dib.2023.109508)
Supplement: Supplementary file 1 [file mmc1.pdf]

COVID 19 IMPACT FOR CHAMPS DSS

📖 Codebook

📖 Data Dictionary Codebook

2021-10-06 09:01:45

#	Variable / Field Name	Field Label<br>*Field Note*	Field Attributes (Field Type, Validation, Choices, Calculations, etc.)
Instrument: **Harmonized Covid19 Impact Questions For Champs Dss** (harmonized_covid19_impact_questions_for_champs_dss)			
1	record_id	Record ID Lakkofsaa Galmeeየ መጠየቅ መለያ	text
2	dss_respondant	RESPONDENT NUMBER lakkofsaa Gaafatamaaየ መላሽ ቁጥር	text
3	dss_hhid	Household DSS IDLakk. DSS Manaaየ ቤት ቁጥር_	text
4	dss_indivdss	Individual DSS ID Lakk. DSS Dhunfaa የግል ቁጥር	text
5	dss_interviewer	Interviewer's name Maqaa gaafilee gaafatuየጠያቂው ስም	text, Identifier
6	dss_intcode	Interviewer's codeKoodi namaa gaafi gaafatuየጠያቂው መለያ ቁጥር	text, Identifier
7	dss_intdate	Interview dateGuyyaa gaafi fi deebin adeemsifameeመጠይቁ የተካሄደበት ቀን	descriptive
8	et_intdate		text (date_dmy)<br>Field Annotation: @ET-DATE
9	dss_intstarttime	Interview start timeyeroo gaafi fi deebin ittii jalqabameeመጠይቁ የተካሄደበት ሰኣት	text (time)
10	dss_instruct	INSTRUCTIONS FOR INTERVIEWER<br>1. INSTRUCTIONS HAVE BEEN PROVIDED IN CAPITAL LETTERS. DO NOT READ THESE INSTRUCTIONS TO RESPONDENTS. 2. READ ALL QUESTIONS AS WRITTEN. DO NOT READ RESPONSES UNLESS INSTRUCTIONS EXPLICITLY STATE TO DO SO. 3. IF QUESTION INSTRUCTIONS STATE TO READ RESPONSE, DO NOT READ "DON'T KNOW" OR "REFUSED TO RESPOND". CIRCLE THESE ONLY IF THEY ARE THE ANSWER GIVEN BY THE RESPONDENTS. 4. INDICATE THE RESPONSE THAT CORRESPONDS TO THE RESPONDENT'S ANSWER.5. FOR OPEN QUESTIONS, ENTER THE RESPONDENT'S ANSWER IN THE SPACE PROVIDED.QAJEELFAMAA NAMA GAAFI GAAFATUF<br>1. QAJEELFAMNII QUBEE GURGUDDAAN FUDHATAMA QABA. QAJEELFAMAA KANAA NAMOOTA GAAFII GAFAII GAAFATAMANIIF HIN DUUBISIIN. 2. GAAFILEE HUNDAA AKKAATA BARRA'EEN DUUBISII. HANGA QAJEELFAMNI ADDAA HI JIRREETTI DEEBILEE HIN DUUBISIIN.3. QAJEELFAMNII AKKA DEEBILEE DUBBISTU AJAJAA TAANAN, HIN BEEKU YOOKIN DEEBISUU DHABUU KAN JEEDHU OSOO HIN DUUBISIN YOO OFII ISAANI JEEDHAN QOFAA GUUTI.4. DEEBII GAAFATAMAAN KAN DEEBISII AGARSISII.5. GAAFILEE BANAA TA'ANIIF, DEEBI DEEBI'EE IDDOO DUWWAA KENNAMEETTI GUUTI<br>መጠይቁን ለሚያካሂደው ሰው መመሪያ<br>1. መመሪያው በ ሆህያት አማራጭ ቀርቡል፡፡ ይህ መመሪያ ለመላሾች አይነበብም፡፡<br>2. ሁሉንም መጠይቅ አንደትቀመጠቅ ይነበብ፡፡ ልዩ ትዛዝ እስካሌለ ድረስ የመልስ አማራጮቹ አይንበቡ፡፡ 3. መጠይቁ አንዲነበብ ልዩ ትዛዝ ካለው ፣ አላውቅም ወይም መመለስ አልፈልግም የሚለውን አማራች አይነበብ፡፡ ከመለሱ ብቻ ይሞላ፡፡ 4. የመላሹ መልስ በትክክል ይቀመጥ፡፡ 5. ክፍት ለሆኑ መጠይቆች፥ የተሰጠውን መልስ በትሰጠው ባዶ ቦታ ይሙሉ	descriptive
11	dss_s1	Section Header: *Socio demographic Characterstics (S)Haala Jirenyaa Haawwasummaa (S)የአኗኗር ሁኔታ መጠይቆች (S)*<br>S1. Age of respondentS1. Umrii hirmaataS1. የመላሹ እድሜ<br>*Age in years**Umurii waggaadhaan**ዕድሜ በአመታት*	text (integer)
12	dss_s2	S2. ResidenceS2. Bakka jireenyaaS2. የመኖርያ ቦታ	radio<br>1 | Urban**Magaala**ከተማ<br>2 | Rural**Baadiyyaa**ገጠር

13	dss_s3	S3. SexS3. SaalaS3. ጾታ	radio<br>1 Male**Dhiira**ወንድ<br>2 Female**Dubara/tii**ሴት
14	dss_s4	S4. Ethnicity S4. Gosa/sanyiiS4. ብሄር	radio<br>1 Oromo**Oromoo**ኦሮሞ<br>2 Amhara**Amaara**አማራ<br>3 Tigre**Tigiree**ትግሬ<br>4 Other**Kan biroo** ሌላ
15	dss_s10_spec_3<br>Show the field ONLY if:<br>[dss_s4] = '4'	Other specify:Kan biraa Ibsii:ሌላ ካለ ይገለጽ:	text
16	dss_s5	S5. Religion S5. AmantaaS5. ሀአይማኖት	radio<br>1 Orthodox**Ortodooksii**ኦርቶዶክስ<br>2 Muslim**Musiliima**ሙስሊም<br>3 Protestant**Pheenxee**ፕሮቴስታንት<br>4 Other**Kan biroo** ሌላ
17	dss_s10_spec_2<br>Show the field ONLY if:<br>[dss_s5] = '4'	Other specify:Kan biraa Ibsii:ሌላ ካለ ይገለጽ:	text
18	dss_s6	S6. Current marrital status S6. Gaa'ilaa yeero ammaa irra jiruuS6. አሁናዊ የጋብቻ ሁኔታ	radio<br>1 Married**Kan fuudhe/heerumte**ያገባ<br>2 Divorced**Kan wal diigaan**ተፋቷል<br>3 Separated**Gargar kan jiraatan**ተለያይቷል<br>4 Widowed**Kan irraa du'e/duutee**መበለት<br>5 Single**Qofaa**ነጠላ
19	dss_s7	S7. Number of family membersS7. Baay'na maati mana tokkoo keessaa waliin jiraatanS7. ቤተሰብ ብዛት<br>*Write numbers**Lakkofsaa kaa'i**በቁር ይቀመጥ*	text (integer)
20	dss_s8	S8. Highest educational levelS8. Sadarkaa baruumsaa guddaan irraa gaahameeS8. የትምርት ደረጃ	radio<br>1 No formal education **Barnoota hawaasa keessatti kennamu**መደበኛ ትምህርት የለም<br>2 Read and write**Dubbisuu fi bareeeessuu**ማንበብ እና መፃፍ<br>3 Primary**Sadarkaa 1ffaa**የመጀመሪያ ደረጃ<br>4 Secondary**Sadarkaa 2ffaa**ሁለተኛ<br>5 College and above **Koolleejjii fi isaa ol**ኮሌጅ እና ከዚያ በላይ
21	dss_s9	S9. Do you have occupation?S9. Hooji qabdaa?S9. ስራ አሎት	radio<br>CH00001 Yes**Eeyyeen **አዎ<br>CH00002 No**Lakki**አይ
22	dss_s10<br>Show the field ONLY if:<br>[dss_s9]='CH00001'	S10. Type of occupationS10. Gosa hooji S10. የስራ አይነት	radio<br>1 House wife**Haadha manaa**የቤት ሚስት<br>2 Governmental employee**Hojjettuu/taa Mootummaa**የመንግስት ሰራተኛ<br>3 Private employee**Hojjetaa/ttuu dhuunfaa**የግል ሰራተኛ<br>4 Farmer**Qonnaan bulaa**ገበሬ<br>5 Other**Kan biroo** ሌላ

<table>
<tr><td>23</td><td>dss_s10_spec

Show the field ONLY if:
[dss_s10] = '5'</td><td>Other specify:Kan biraa Ibsii:ሌላ ካለ ይገለጽ:</td><td>text</td></tr>
<tr><td>24</td><td>dss_s11</td><td>S11. How much is your household Monthly income? (In Ethiopian birr) S11. Galii ji'atti argamuu meeqa? (Qarshii Itoophiyaatin))S11. የቤተሰብ ወርሀዊ ገቢ ስንት ነው?
Mention in ETB**Qarshii Itoophiyaatin kaa'i**በኢትዮጲያ ብር ይቀመጥ</td><td>text</td></tr>
<tr><td>25</td><td>dss_s12</td><td>S12. Do you have health insurance?S12. Baraarsa fayyaa qabdaa? S12. የጤና መድን አሎት??</td><td>radio<table>
<tr><td>CH00001</td><td>Yes**Eeyyeen **አዎ</td></tr>
<tr><td>CH00002</td><td>No**Lakki**አይ</td></tr>
</table></td></tr>
<tr><td>26</td><td>dss_v1</td><td>Section Header: INTERVIEWER TO PARTICIPANT: I would like to ask the head of the household some questions about coronavirus or COVID-19.SECTION 1Nama gaafatu gara hirmaataa/tuutti:: Dursaa/hoogganaa mana kanaa waa'ee koronaa vaayirasii gaaffii muraasa akka naaf deebisaniif kabajaanan gaafadhaለተሳታፊ ቃለ-መጠይቅ: - ስለ ኮሮናቫይረስ ወይም ስለ COVID-19 አንዳንድ ጥያቄዎችን ለቤተሰቡ ዋና ኃላፊ መጠየቅ እፈልጋለሁ

V1. Have you heard about the coronavirus/COVID-19?V1. Waa'ee Koronaa Vaayirasii dhageessee jirtaa?V1. እርስዎ ስለ COVID-19/ coronavirus ያለውን ሰምተዋል?</td><td>radio<table>
<tr><td>CH00001</td><td>Yes**Eeyyeen **አዎ</td></tr>
<tr><td>CH00002</td><td>No**Lakki**አይ</td></tr>
<tr><td>98</td><td>Don't know**Hin beeku**አላውቅም</td></tr>
<tr><td>99</td><td>Refused to respond**Deebiif eeyyamamaa tahuu dhabuu**ለመመለስ ፈቃደኛ አልሆነም/ችም</td></tr>
</table></td></tr>
<tr><td>27</td><td>dss_v2

Show the field ONLY if:
[dss_v1] = 'CH00001'</td><td>V2. To your knowledge, which of the following measures can you adopt to reduce the risk of contracting coronavirus/COVID-19?
(PLEASE DO NOT READ, CHECK MULTIPLE RESPONSES THAT APPLY, OR WRITE RESPONSE FOR "OTHER" IF SELECTED)V2. Akka beekumsakeetti,dhiibbaa koronaa vaayirasiin geessisu hir'isuuf kan armaan gadii keessa kam fayyadamaa jirta?
(MAALO HIN DUUBISINIIF, DEEBI TOKKOO OL JIRAA TAANAN ILAALI, YOOKIN KAN BIRAA KAN JEEDHU GUUTI YOO DEEBI'E JIRAATE)V2.እርስዎ እንደሚያውቁት የኮሮናቫይረስ / COVID-19 የመያዝ አደጋን ለመቀነስ ከሚከተሉት እርምጃዎች ውስጥ የትኛውን መውሰድ ይችላሉ? (አይነበብ፣ ሁሉም የተመረጡ መልሶች ይቀመጡ)</td><td>checkbox<table>
<tr><td>1</td><td>dss_v2___1</td><td>Handwashing**Harka dhiqannaa**እጅን መታጠብ</td></tr>
<tr><td>2</td><td>dss_v2___2</td><td>Sanitizer Use**Sanitaayizerii fayyadamuu**የንፅህና አጠባበቅ አጠቃቀም</td></tr>
<tr><td>3</td><td>dss_v2___3</td><td>Avoid handshake/physical greeting**Harka wal qabachuu dhiisuu**እጅ መጨባበጥ / አካላዊ ሰላምታን ያስወግዱ</td></tr>
<tr><td>4</td><td>dss_v2___4</td><td>Mask use**Haguugduu Fayyadamuu: **ጭምብልን መጠቀም</td></tr>
<tr><td>5</td><td>dss_v2___5</td><td>Gloves use**Gaantii/Uffata harkaa**ጓንት መጠቀም</td></tr>
<tr><td>6</td><td>dss_v2___6</td><td>Avoid travel**Imala Hambisuu**ጉዞን ያስወግዱ</td></tr>
<tr><td>7</td><td>dss_v2___7</td><td>Staying at home**Mana keessa turuu**እንደተረጋገጠ ላይ ቤት</td></tr>
<tr><td>8</td><td>dss_v2___8</td><td>Avoid going out unless necessary**Dhimma barbaachisurraa kan hafe ala bahuu dhiisuudhaan**እንዳትታለሉ በመሄድ ውጭ በስተቀር አስፈላጊ</td></tr>
<tr><td>9</td><td>dss_v2___9</td><td>Avoid crowded places**Bakka baay'ee namni itti walitti qabamu dhorkuun**የተጨናነቁ ቦታዎችን ያስወግዱ</td></tr>
<tr><td>10</td><td>dss_v2___10</td><td>Keep ~2 meters space between yourself and others**Ofiikeefi namoota kanneen biroo 2m tahu fagaachuun**በእራስዎ እና በሌሎች መካከል ~ 2 ሜትር ቦታን ይጠብቁ</td></tr>
<tr><td>88</td><td>dss_v2___88</td><td>Other**Kan biroo **ሌላ ካለ ይግለጹ</td></tr>
<tr><td>98</td><td>dss_v2___98</td><td>Don't know**Hin beeku**አላውቅም</td></tr>
<tr><td>99</td><td>dss_v2___99</td><td>Refused to respond**Deebiif eeyyamamaa tahuu dhabuu**ለመመለስ ፈቃደኛ አልሆነም/ችም</td></tr>
</table></td></tr>
</table>

28	dss_v2handother<br><br>Show the field ONLY if:<br>[dss_v2(88)] = '1'	Other specify:Kan biraa Ibsii:ሌላ ካለ ይገለጽ:	text
29	dss_v3<br><br>Show the field ONLY if:<br>[dss_v1] = 'CH00001'	V3. What steps has your community/government taken to curb the spread of the coronavirus in your area?<br>(PLEASE READ ALOUD, SELECT ALL THAT APPLY) V3. Bakka jiraatutti hawaasni /Mootummaan tatamsa'ina koronaa Vaayirasii hir'isuuf waantoonni godhaman maal fa'i?<br>(MAALOO SAGALEE OL KAASI DUUBISIF, DEEBI FILATAMEE HUNDAA GUUTI)V3.በእርስዎ አካባቢ የ coronavirus ስርጭት ን መግታት የማህበረሰብ / መንግስት ምን እርምጃዎች ይወሰዳል?<br>(እባኮ ጮክ ብለው ያንብቡላቸውና ሁሉንም መልስ አካት)	checkbox<br>00 | dss_v3___00 | None**hin jiru**የለም<br>01 | dss_v3___01 | Advised citizens to stay at home**Mana keessa akka turaniif gorsa lammiileef kennameera**ዜጎች በቤት እንዲቆዩ የተመከሩ<br>02 | dss_v3___02 | Advised to avoid gatherings**Bakka baay'ee namni itti walitti qabamu dhorkuun**ሰው ሚበዛበት ቦታ ክልከላ<br>03 | dss_v3___03 | Restricted travel within country/area** Imala biyyoolessaa dangessuun**በሀገር ውስጥ የተከለከለ ጉዞ<br>04 | dss_v3___04 | Restricted international travel**Imalaa biyyaa alaa daangessuu**ከሀገር ዉጭ ጉዞ ክልከላ<br>05 | dss_v3___05 | Closure of schools and universities**Manneen barnoota fi Yuunivarsiitiiwwan cufuun**ትምህርት ቤቶች እና ዩኒቨርስቲዎች መዘጋት<br>06 | dss_v3___06 | Curfew/lockdown**Dhorkaa sosochii**የእንቅስቃሴ እገዳ/ክልከላ<br>07 | dss_v3___07 | Closure of non-essential businesses**Manneen hojii hangas maraa hin barbaachifnee cufuun** አስፈላጊ ያልሆኑ ንግድ ቤቶች መዘጋት<br>08 | dss_v3___08 | Sensitization/public awareness**Hubannoon ummataaf kennameera**የንቃተ-ህሊና / የህዝብ ግንዛቤ<br>09 | dss_v3___09 | Established isolation centers**Giddu gala qofaa ta'e qopheessuun**የተገለሉ ሚቆዩበት ማዕከሎች ተቋቁመዋል<br>10 | dss_v3___10 | Disinfection of public places**Bakkeewwan Uummanni baay'inaan walitti qabamu Faalamaan ala gochuun ** ይፋዊ ቦታዎችን በኬሚካል ማጽዳት<br>88 | dss_v3___88 | Other**Kan biroo **ሌላ ካለ ይግለጹ<br>98 | dss_v3___98 | Don't know**Hin beeku**አላውቅም<br>99 | dss_v3___99 | Refused to respond**Deebiif eeyyamamaa tahuu dhabuu**ለመመለስ ፈቃደኛ አልሆነም/ችም
30	dss_v3other<br><br>Show the field ONLY if:<br>[dss_v3(88)] = '1'	Other specify:Kan biraa Ibsii:ሌላ ካለ ይገለጽ:	text

31	dss_v4<br><br>Show the field ONLY if:<br>[dss_v1] = 'CH00001'	V4. Were any of your household members tested for COVID-19?V4. Qorannoon Koranaa vaayirasii miseensa maatiikee keessa kan godhameef ni jiraa?V4.ማንኛውም የ ቤተሰብ አባላት መካከል ለ COVID-19 የተመረመረ ነበር ?	radio<br>CH00001 | Yes**Eeyyeen **አዎ<br>CH00002 | No**Lakki**አይ<br>98 | Don't know**Hin beeku**አላውቅም<br>99 | Refused to respond**Deebiif eeyyamamaa tahuu dhabuu**ለመመለስ ፈቃደኛ አልሆነም/ችም
32	dss_v5<br><br>Show the field ONLY if:<br>[dss_v4] = 'CH00001'	V5. Did any of your household members test positive for COVID-19? v5. Qorannoon Koranaa vaayirasiif miseensa maatiikee keessa kan poozitiivii tahe ni jiraa? V5. በማንኛውም ከእርስዎ ቤተሰብ<br>አባላት በምርመራ COVID- 19 የተገኘበት ነበር?	radio<br>CH00001 | Yes**Eeyyeen **አዎ<br>CH00002 | No**Lakki**አይ<br>98 | Don't know**Hin beeku**አላውቅም<br>99 | Refused to respond**Deebiif eeyyamamaa tahuu dhabuu**ለመመለስ ፈቃደኛ አልሆነም/ችም
33	dss_p1	Section Header: *Food availability (P)INTERVIEWER TO PARTICIPANT: Getting enough food can also be a problem for some households due to coronavirus or COVID-19. I would like to ask you some questions about food availability in your household. Waa'ee haala nyaata jiraachu fi dhabuu waliin kan walqabate (P) Waa'ee nyaata irratti gaaffilee muraasa isiiin gaafachuun barbaadaየአመጋገብ ባህሪዎች (P) በቤተሰብዎ ውስጥ ስላለው የምግብ አቅርቦት አንዳንድ ጥያቄዎችን ልጠይቅዎት እፈልጋለሁ*<br><br>P1a. Before mid-March 2020, did it ever happen that your household did not have enough food to eat? P1a. Giddu gala Bitootessa 2012 dura,Maatiinkee Soorata Soorachuuf nyaatni gahaa hin turree? P1a. ከመጋቢት 2020-አጋማሽ በፊት, በቤተሰቦ ውስጥ የሚበላ ምግብ ጠቶ ያውካል?	radio<br>CH00001 | Yes**Eeyyeen **አዎ<br>CH00002 | No**Lakki**አይ<br>98 | Don't know**Hin beeku**አላውቅም<br>99 | Refused to respond**Deebiif eeyyamamaa tahuu dhabuu**ለመመለስ ፈቃደኛ አልሆነም/ችም
34	dss_p1b<br><br>Show the field ONLY if:<br>[dss_p1] = 'CH00001'	P1b. How many times did your household not have enough food to eat?<br><br>ENTER 98 IF "DON'T KNOW".<br>ENTER 99 IF "REFUSED TO ANSWER".P1b. Yeero meeqaf nyaata dhabdan? Hin beeku taanan 98<br>Deebisuuf eeyyamaa miti taanan 99 galchiiP1b. ለምንያሂል ግዜ ቤት ዉስጥ ምግብ ጠፍቶ ነበር? አታውቅም... 98<br>አሻፈረኝ ወደ ምላሽ ----- 99	text

<table>
<tr><td>35</td><td>dss_p1c

Show the field ONLY if:
[dss_p1] = 'CH00001'</td><td>P1c. Why did your household not have enough to eat or not what you wanted to eat before mid-March 2020?
(PLEASE READ ALOUD, SELECT ALL THAT APPLY)P1c. Bitootessa 2012 'n duraa Maalif ture kan nyaata gahaa/barbaaddan argachuun isiin dhibe?
(MAALOO SAGALEE OL KAASI DUUBISIF, DEEBI FILATAMEE HUNDAA GUUTI)P1c. ከመጋቢት 2020 አጋማሽ ጀምሮ ለምን ነበር ምግብ ያጣችሁት?
(እባኮ ጮክ ብለው ያንብቡላቸውና ሁሉንም መልስ አካት)</td><td>checkbox
<table>
<tr><td>01</td><td>dss_p1c___01</td><td>Couldn't afford to buy more food**Nyaata bituuf qarshii gahaa argachuu hin dandeenyeef**ተጨማሪ ምግብ ለመግዛት አቅም አልነበረኝም</td></tr>
<tr><td>02</td><td>dss_p1c___02</td><td>Couldn't get out to buy food**Nyaata bituuf ala vahuu hin dandeenye**ምግብ ለመግዛት መውጣት አልተቻለም</td></tr>
<tr><td>03</td><td>dss_p1c___03</td><td>Afraid to go or didn't want to go out to buy food**Nyaata bituuf ala bahuu sodaachuu yookiin fedhii ala bahuu dhabuu**ወቶ መግዛት ፈራሁ/አልወጣሁም</td></tr>
<tr><td>04</td><td>dss_p1c___04</td><td>Couldn't get groceries or meals delivered to me**Mana nyaataa kan nyaata naaf dhiyeesu argachuu dhabuu** አሳልፈጊውን ሸቀጥ ወይም ምግብ ወደ እኔ የሚያመጣ ለማግኘት አልተቻለም</td></tr>
<tr><td>05</td><td>dss_p1c___05</td><td>Stores didn't have the food I wanted**Nyaatan barbaade Warri gurguran dhabuu**መደብሮች ውስጥ እኔ ምፈልገው ምግብ አልነበረም</td></tr>
<tr><td>88</td><td>dss_p1c___88</td><td>Other**Kan biroo **ሌላ ካለ ይግለጹ</td></tr>
<tr><td>98</td><td>dss_p1c___98</td><td>Don't know**Hin beeku**አላውቅም</td></tr>
<tr><td>99</td><td>dss_p1c___99</td><td>Refused to respond**Deebiif eeyyamamaa tahuu dhabuu**ለመመለስ ፈቃደኛ አልሆነም/ችም</td></tr>
</table></td></tr>
<tr><td>36</td><td>dss_p2other

Show the field ONLY if:
[dss_p1c(88)] = '1'</td><td>Other specify:Kan biraa Ibsii:ሌላ ካለ ይገለጽ:</td><td>text</td></tr>
<tr><td>37</td><td>dss_p2a</td><td>P2a. Since the beginning of the COVID lockdown, has it happened that your household did not have enough food to eat?P2a. Akkuma jalqaba koronaan vaayirasii mudateen,maatiin ati waliin jiraattu keessa Soorata gahaa Sooratamuu hin qaban turanii?P2a. በመጀመሪያ COVID መዝጋት ቀን ጀምሮ, የእርስዎ ቤተሰብ በቂ ምግብ አጥቶ ያውቃል?</td><td>radio
<table>
<tr><td>CH00001</td><td>Yes**Eeyyeen **አዎ</td></tr>
<tr><td>CH00002</td><td>No**Lakki**አይ</td></tr>
<tr><td>98</td><td>Don't know**Hin beeku**አላውቅም</td></tr>
<tr><td>99</td><td>Refused to respond**Deebiif eeyyamamaa tahuu dhabuu**ለመመለስ ፈቃደኛ አልሆነም/ችም</td></tr>
</table></td></tr>
<tr><td>38</td><td>dss_p2b

Show the field ONLY if:
[dss_p2a] = 'CH00001'</td><td>P2b. During this time, how many times did your household not have enough food to eat?

ENTER 98 IF "DON'T KNOW".
ENTER 99 IF "REFUSED TO ANSWER". P2b. Akkuma jalqaba koronaan vaayirasii mudateen,maatiin ati waliin jiraattu keessa Soorata gahaa Sooratamuu hin qaban turanii?
Hin beeku taanan 98 galchi
Deebisuuf eeyyamaa miti taanan 99 galchiiP2b. በዚህ ወቅት, የእርስዎ ቤተሰብ ለስንት ጊዜ በቂ ምግብ የላቸውም ነበር? አላውቅም... 98 አሻፈረኝ 99 አስገባ</td><td>text</td></tr>
</table>

<table>
<tr><td>39</td><td>dss_p3

Show the field ONLY if:
[dss_p2a] = 'CH00001'</td><td>P3. Why did your household not have enough to eat (or not what you wanted to eat) since mid-March 2020? (SELECT ALL THAT APPLY)P3. Giddu gala bitootessa 2012,Maatiinkee Maaliif Soorataa gahaa soorachuu dhabani?P3. ከመጋቢ 2020 አጋማሽ ጀምሮ ለምን ነበር የእርስዎ ቤተሰብ በቂ የምበላው ምግብ (የሚፈልገውን ምግብ) ያጣው?</td><td>checkbox<table>
<tr><td>01</td><td>dss_p3___01</td><td>Couldn't afford to buy more food**Nyaata bituuf qarshii gahaa argachuu hin dandeenyeef**ተጨማሪ ምግብ ለመግዛት አቅም አልነበረኝም</td></tr>
<tr><td>02</td><td>dss_p3___02</td><td>Couldn't get out to buy food**Nyaata bituuf ala vahuu hin dandeenye**ምግብ ለመግዛት መውጣት አልተቻለም</td></tr>
<tr><td>03</td><td>dss_p3___03</td><td>Afraid to go or didn't want to go out to buy food**Nyaata bituuf ala bahuu sodaachuu yookiin fedhii ala bahuu dhabuu**ወቶ መግዛት ፈራሁ/አልወጣሁም</td></tr>
<tr><td>04</td><td>dss_p3___04</td><td>Couldn't get groceries or meals delivered to me**Mana nyaataa kan nyaata naaf dhiyeesu argachuu dhabuu** አሳልፈጊውን ሸቀጥ ወይም ምግብ ወደ እኔ የሚያመጣ ለማግኝት አልተቻለም</td></tr>
<tr><td>05</td><td>dss_p3___05</td><td>Stores didn't have the food I wanted**Nyaatan barbaade Warri gurguran dhabuu**መደብሮች ውስጥ እኔ ምፈልገው ምግብ አልነበረም</td></tr>
<tr><td>88</td><td>dss_p3___88</td><td>Other**Kan biroo **ሌላ ካለ ይግለጹ</td></tr>
<tr><td>98</td><td>dss_p3___98</td><td>Don't know**Hin beeku**አላውቅም</td></tr>
<tr><td>99</td><td>dss_p3___99</td><td>Refused to respond**Deebiif eeyyamamaa tahuu dhabuu**ለመመለስ ፈቃደኛ አልሆነም/ችም</td></tr>
</table></td></tr>
<tr><td>40</td><td>dss_p4other

Show the field ONLY if:
[dss_p3(88)] = '1'</td><td>Other specify:Kan biraa Ibsii:ሌላ ካለ ይገለጽ:</td><td>text</td></tr>
</table>

41	dss_m1	Section Header: *COVID-19 related shocks/coping (M)INTERVIEWER TO PARTICIPANT: Now I would like to ask you about events that may have affected your household since mid-March.Kooviidii-19 wal qabatee wantoota nama Naasisan (M) Namni gaafatu hirmaattotaaf yoo ibsu:Amma waantoota Giddu gala bitootessaa keessa warra waliin galtan mudate/miidhe si gaafachuun barbaadaCOVID-19 ተዛማጅ ድንጋጤዎች / መቋቋም (M) ጠያቂ ወደ ተሳታፊ: ከመጋቢት 2020 አጋማሽ ጀምሮ ቤተሰቦ ላይ የደረሱ ጉዳቶችን የተመለከቱ መጠይቆች*<br><br>M1. Has your household been affected by any of these events since mid-March? (PLEASE READ ALOUD AND SELECT ALL THAT APPLY)M1. Warri waliin galtan wantoota kanaan miidhamaniiruu yeroo giddu gala bitootessaa?<br>(MAALOO SAGALEE OL KAASI DUUBISIF, DEEBI FILATAMEE HUNDAA GUUTI)M1. በእርስዎ ቤተሰብ ዉስጥ ከመጋቢት አጋማሽ ጀምሮ ማንኛው ተጽዕኖ ወይም ክስተቶች ነበር? (ድምጽ ከፍ ብሎ ይነበብና ሁሉም መልስ ይካተት)	checkbox<br>01 dss_m1___01 Job loss**Hojii dhabinsa**የሥራ ማጣት<br>02 dss_m1___02 Nonfarm business closure**Daldalli qonna hin taane cuufamuu ** ከ እርሻ ውጭ ያሉ ንግድ መዘጋት<br>03 dss_m1___03 Disruption of farming**Qonni dhaabachuu/gargar cituu**የእርሻ መቋረጥ<br>04 dss_m1___04 Disruption of livestock activities**Hojiin Horii Horsiisuun/furdisuu addaan cituu**የእንሰሳት ሥራዎች መቋረጥ<br>05 dss_m1___05 Disruption of fishing activities**Hojiin Qurxummii qabuun addaan cituu**የዓሣ ማጥመድ ሥራዎች መቋረጥ<br>06 dss_m1___06 Increased price of farming or business inputs**Gatiin Oomisha Qonnaa yookiin bu'aan daldalaa ol ka'uu**የግብርና ዋጋ ወይም የንግድ ግብዓቶች መጨመር<br>07 dss_m1___07 Decreased price of farming or business outputs**Gatiin Oomisha Qonnaa yookiin bu'aan daldalaa gadi bu'uu**የግብርና ዋጋ ወይም የንግድ ውጤቶች ቅናሽ<br>08 dss_m1___08 Increased price of major food items consumed**Gatiin nyaata/miidhama Qaala'uu**የዋና የምግብ ዋጋ ጨምሯል<br>09 dss_m1___09 Illness, injury, or death of any household member**Dhukkuba,balaa,yookiin duuni miseensa maatii keessaa kan mudate yoo jiraate**ማንኛውንም ሕመም, ጉዳት, ወይም ሞት የቤተሰብ አባል<br>88 dss_m1___88 Other major problem** Kan biroo midhamni guddaan yoo jiraate **ሌሎች ዋና ዋና ተጽዕኖ ችግሮች / ክስተቶች ካሉ<br>10 dss_m1___10 Not affected by any listed or other major problems/events** Kan tarrefaman yookin kan biroo kaamiinuu hin miidhamne** ማንኛውም ከተዘረዘሩት ወይም በሌሎች ዋና ዋና ተጽዕኖ ችግሮች / ክስተቶች አልተጎዳሁም<br>98 dss_m1___98 Don't know**Hin beeku**አላውቅም<br>99 dss_m1___99 Refused to respond**Deebiif eeyyamamaa tahuu dhabuu**ለመመለስ ፈቃደኛ አልሆነም/ችም
42	dss_m1other<br><br>Show the field ONLY if:<br>[dss_m1(88)] = '1'	Other specify:Kan biraa Ibsii:ሌላ ካለ ይገለጽ:	text
43	dss_m2<br><br>Show the field ONLY if:<br>[dss_m1(01)] = '1' or [dss_m1(02)] = '1' or [dss_m1(03)] = '1'	M2. How did your household cope with these difficulties encountered since mid-March?<br>(PLEASE DO NOT READ, SELECT ALL THAT APPLY)M2. Rakkina giddu gala bitootessaatti qunname maatinkee akkamiin	checkbox<br>00 dss_m2___00 Did nothing**Homaa hin hojjenne**ምንም አላደረገም

	or [dss_m1(04)] = '1' or [dss_m1(05)] = '1' or [dss_m1(06)] = '1' or [dss_m1(07)] = '1' or [dss_m1(88)] = '1' or [dss_m1(98)] = '1'	dabarsan?<br>(MAALOO hin duubisiin, DEEBI FILATAMEE HUNDAA GUUTI)M2. ከመጋቢት አጋማሽ አንስቶ ያጋጠሟቸውን እነዚህን ችግሮችን ቤተሰቦችዎ እንዴት ተቋቋሙ?<br>(እባክዎ አያነቡ ፣ ለሚመለከታቸው ሁሉ ይምረጡ)	01	dss_m2___01	Sale of assets**Qabeenya gurguruun**የንብረት ሽያጭ
			02	dss_m2___02	Engaged in additional income generating activities**Wantoota galii dabalataa maddisiisan keessa galuun.** በተጨማሪ የገቢ ማስገኛ ሥራዎች ተሰማርቷል
			03	dss_m2___03	Assistance from friends & family**Gargaarsa hiriyaa fi maatiitiin**ከጓደኞች እና ከቤተሰብ የሚደረግ ድጋፍ
			04	dss_m2___04	Borrowed from friends & family**Hiriyootaa fi maatiirraa liqeeffachuun**ከጓደኞች እና ከቤተሰብ ተበድረው
			05	dss_m2___05	Took a Loan**Liqii fudhachuun**ብድር ወስደዋል
			06	dss_m2___06	Delayed payment obligations**Kafaltii turtiidhaan deebi'u fudhachuun**የክፍያ ግዴታዎች ማዘግየት
			07	dss_m2___07	Sold harvest in advance**Makara sassabame gurguruun**የተሰበሰበውን መከር/እህል መሸጥ
			08	dss_m2___08	Reduced food Consumption**Hamma nyaata nyaatani hir'isuudhaan** የምግብ ፍጆታ መቀነስ
			09	dss_m2___09	Reduced nonfood Consumption**Wantoonni kan biroo kaffaltii garaa garaa qaban gadi bu'uun**ምግብ ያልሆነ ፍጆታ መቀነስ
			10	dss_m2___10	Relied on Savings**Qusannoorraatti hirkachuun**በቁጠባዎች ላይ መመስረተ
			11	dss_m2___11	Received assistance from NGO**Gargaarsa dhaabbata miti -mootummaarraa fudhachuun**ከ NGO / ድርጅቶች ድጋፍ ተቀበልኩ
			12	dss_m2___12	Took advanced payment from employer**Mindessaarraa kaltii fudhachuun**ከአሰሪ የማካካሻ ክፍያ ወስዷል
			13	dss_m2___13	Received assistance from government**Gargaarsa mootummaarraa fudhachuun**ከመንግስት የተገኘ እርዳታ
			88	dss_m2___88	Other**Kan biroo **ሌላ ካለ ይግለጹ
			98	dss_m2___98	Don't know**Hin beeku**አላውቅም
			99	dss_m2___99	Refused to respond**Deebiif eeyyamamaa tahuu dhabuu**ለመመለስ ፈቃደኛ አልሆነም/ችም

44	dss_m2other<br><br>Show the field ONLY if:<br>[dss_m2(88)] = '1'	Other specify:Kan biraa Ibsii:ሌላ ካለ ይገለጽ:	text
45	dss_q1	Section Header: *Under-five child healthcare services (Q)INTERVIEWER TO PARTICIPANT: Getting adequate health services can be a problem due to coronavirus or COVID-19. I would like to ask some questions about the children under age 5 years in your household.Tajaajila kunuunsa Fayyaa ijoollota waggaa shanii gadiif (Q) NAMNI GAAFATU HIRMAATTOTAAF YOO IBSU: : Getting adequate health services can be a problem due to coronavirus or COVID-19.Waa'ee ijoollota waggaa shanii gadi mana tokko keessa jirattaniirratti gaaffii muraasa si gaafachuun barbaada*ከአምስት ዓመት በታች የሆኑ የህፃናት ጤና አጠባበቅ አገልግሎቶች *(Q)* ለተሳታፊ ቃለ-መጠይቅ-በቂ የጤና አገልግሎቶችን ማግኘት በኮሮቫይረስ ወይም በ *COVID-19* ምክንያት ችግር ሊሆን ይችላል ፡ ከቤተሰብዎ ዕድሜያቸው ከ *5* ዓመት በታች ለሆኑ ሕፃናት አንዳንድ ጥያቄዎችን መጠየቅ እፈልጋለሁ ፡<br><br>Q1. Since mid-March, did any of the children under age 5 attend any healthcare visits?Q1. Yeroo giddu gala bitootessaatti,Daa'ma waggaa Shanii gadii kamiyyuu haa tahu kan tajaajila fayyaf kilinika deemee argate ni jiraa?Q1. ልጁ ምን ዓይነት የሕክምና እንክብካቤ አግኝቷል ? (እባክዎ አያነቡ ፣ ለሚመለከታቸው ሁሉ ይምረጡ)	radio<br>CH00001 — Yes**Eeyyeen **አዎ<br>CH00002 — No**Lakki**አይ<br>98 — Don't know**Hin beeku**አላውቅም<br>99 — Refused to respond**Deebiif eeyyamamaa tahuu dhabuu**ለመመለስ ፈቃደኛ አልሆነም/ችም
46	dss_q2<br><br>Show the field ONLY if:<br>[dss_q1] = 'CH00001'	Q2. What kind of medical care did the child receive?<br>(PLEASE DO NOT READ, SELECT ALL THAT APPLY)Q2. Daa'imni gosa tajaajila fayyaa kam fudhate?<br>(MAALOO hin duubisiin, DEEBI FILATAMEE HUNDAA GUUTI)Q2. ከመጋቢት አጋማሽ አንስቶ ለህፃን ወይም ለልጅ የህክምና እንክብካቤ ወይም ክሊኒክ ጉብኝት የሚፈልጉበት ጊዜ ግን አልነበረም? (እባክዎ አያነቡ ፣ ለሚመለከታቸው ሁሉ ይምረጡ)	checkbox<br>01 — dss_q2___01 — Routine follow-up visits for kids**Beellama itti fufinsa daa'immaniif godhamuuf**ለልጆች መደበኛ ክትትል ጉብኝቶች<br>02 — dss_q2___02 — Routine vaccinations**Beellama Talaallii itti fufinsaaf**መደበኛ ክትባቶች<br>03 — dss_q2___03 — Malaria treatment**Yaalii dhukkuba busaaf**የወባ ህክምና<br>04 — dss_q2___04 — HIV treatment**Yaalii HIV**የኤች አይ ቪ ህክምና<br>05 — dss_q2___05 — Clinic visits for any illness**Dhukkuba kamiifuu yoo kilinka deemeeras tahe haa tahu**ለማንኛውም ህመም ክሊኒክ መጎብኘት<br>06 — dss_q2___06 — Services for malnutrition**Tajaajila hanqina nyaataa argachuuf**ለተመጣጠነ ምግብ እጥረት የሚረዱ አገልግሎቶች<br>88 — dss_q2___88 — Other**Kan biroo **ሌላ ካለ ይግለጹ<br>98 — dss_q2___98 — Don't know**Hin beeku**አላውቅም<br>99 — dss_q2___99 — Refused to respond**Deebiif eeyyamamaa tahuu dhabuu**ለመመለስ ፈቃደኛ አልሆነም/ችም
47	dss_q2other<br><br>Show the field ONLY if:<br>[dss_q2(88)] = '1'	Other specify:Kan biraa Ibsii:ሌላ ካለ ይገለጽ:	text
48	dss_q3	Q3. Since mid-March, was there a time you needed medical care or clinic visit for a baby or child but could not do so?Q3. Yeroo giddu gala bitootessaa,Yeroon ati tajaajila fayyaa yookiin beellama kilinikaa daa'imakeef barbaadde ni jira turee,garuu gochuufi kan hin dandeenye?Q3. በዚህ ወቅት ስንት የሕክምና እንክብካቤ ወይም ክሊኒካዊ ጉብኝቶች አምልጠዋል?	radio<br>CH00001 — Yes**Eeyyeen **አዎ<br>CH00002 — No**Lakki**አይ<br>98 — Don't know**Hin beeku**አላውቅም<br>99 — Refused to respond**Deebiif eeyyamamaa tahuu dhabuu**ለመመለስ ፈቃደኛ አልሆነም/ችም

<table>
<tr><td>49</td><td>dss_q3a

Show the field ONLY if:
[dss_q3]='CH00001'</td><td>Q3a. During this time, how many medical care or clinical visits were missed?Q3. Yeroo giddu gala bitootessaa,Yeroon ati tajaajila fayyaa yookiin beellama kilinikaa daa'imakeef barbaadde ni jira turee,garuu gochuufi kan hin dandeenye?
Q3a. በዚህ ወቅት ስንት
የሕክምና እንክብካቤ ወይም ክሊኒካዊ ጉብኝቶች አምልጠዋል?</td><td>text</td></tr>
<tr><td>50</td><td>dss_q4

Show the field ONLY if:
[dss_q3] = 'CH00001'</td><td>Q4. What kind of medical care did the child need but did not receive?
(PLEASE DO NOT READ, SELECT ALL THAT APPLY)Q4. Gosa tajaajila fayyaa daa'imnikee barbaade garuu hin fudhatiin hafe maali?
(MAALOO HIN DUUBISN, DEEBI FILATAMEE HUNDAA GUUTI))Q4. ህፃኑ ምን ዓይነት የህክምና አገልግሎት ፈለገ ግን አላገኘም? (እባክዎ አያነቡ ፣ ለሚመለከታቸው ሁሉ ይምረጡ)</td><td>checkbox<table>
<tr><td>01</td><td>dss_q4___01</td><td>Routine follow-up visits for kids**Beellama itti fufinsa daa'immaniif godhamuuf**ለልጆች መደበኛ ክትትል ጉብኝቶች</td></tr>
<tr><td>02</td><td>dss_q4___02</td><td>Routine vaccinations**Beellama Talaallii itti fufinsaaf**መደበኛ ክትባቶች</td></tr>
<tr><td>03</td><td>dss_q4___03</td><td>Malaria treatment**Yaalii dhukkuba busaaf**የወባ ህክምና</td></tr>
<tr><td>04</td><td>dss_q4___04</td><td>HIV treatment**Yaalii HIV**የኤች አይ ቪ ህክምና</td></tr>
<tr><td>05</td><td>dss_q4___05</td><td>Clinic visits for any illness**Dhukkuba kamiifuu yoo kilinka deemeeras tahe haa tahu**ለማንኛውም ህመም ክሊኒክ መጎብኘት</td></tr>
<tr><td>06</td><td>dss_q4___06</td><td>Services for malnutrition**Tajaajila hanqina nyaataa argachuuf**ለተመጣጠነ ምግብ እጥረት የሚረዱ አገልግሎቶች</td></tr>
<tr><td>88</td><td>dss_q4___88</td><td>Other**Kan biroo **ሌላ ካለ ይግለጹ</td></tr>
<tr><td>98</td><td>dss_q4___98</td><td>Don't know**Hin beeku**አላውቅም</td></tr>
<tr><td>99</td><td>dss_q4___99</td><td>Refused to respond**Deebiif eeyyamamaa tahuu dhabuu**ለመመለስ ፈቃደኛ አልሆነም/ችም</td></tr>
</table></td></tr>
<tr><td>51</td><td>dss_q4other

Show the field ONLY if:
[dss_q4(88)] = '1'</td><td>Other specify:Kan biraa Ibsii:ሌላ ካለ ይገለጽ:</td><td>text</td></tr>
<tr><td>52</td><td>dss_q5

Show the field ONLY if:
[dss_q3] = 'CH00001'</td><td>Q5. Why did your child not receive healthcare?
(PLEASE DO NOT READ, SELECT ALL THAT APPLY)Q5. Daa'imnikee Tajaajila fayyaa maaliif hin fudhanne?
(MAALOO HIN DUUBISN, DEEBI FILATAMEE HUNDAA GUUTI)Q5. ልጅዎ ለምን የጤና እንክብካቤ አላደረገም ? (እባክዎ አያነቡ ፣ ለሚመለከታቸው ሁሉ ይምረጡ)</td><td>checkbox<table>
<tr><td>01</td><td>dss_q5___01</td><td>Clinic closed**kiliniki cufaa ture**ክሊኒክ ተዘግቷል</td></tr>
<tr><td>02</td><td>dss_q5___02</td><td>Out of vaccines or medications**Talaallii yookiin Qorichaan ala**ከክትባቶች ወይም መድሃኒቶች ዉጭ</td></tr>
<tr><td>03</td><td>dss_q5___03</td><td>Did not get transportation**Geejjiba hin arganne**መጓጓዣ አላገኘም</td></tr>
<tr><td>04</td><td>dss_q5___04</td><td>Lockdown**Dhorkinsa**ክልከላ</td></tr>
<tr><td>05</td><td>dss_q5___05</td><td>Scared to go**Deemuu Sodaachuu**ለመሄድ ፈርቷል</td></tr>
<tr><td>88</td><td>dss_q5___88</td><td>Other**Kan biroo **ሌላ ካለ ይግለጹ</td></tr>
<tr><td>98</td><td>dss_q5___98</td><td>Don't know**Hin beeku**አላውቅም</td></tr>
<tr><td>99</td><td>dss_q5___99</td><td>Refused to respond**Deebiif eeyyamamaa tahuu dhabuu**ለመመለስ ፈቃደኛ አልሆነም/ችም</td></tr>
</table></td></tr>
</table>

53	dss_q5other<br>Show the field ONLY if:<br>[dss_q5(88)] = '1'	Other specify:Kan biraa Ibsii:ሌላ ካለ ይገለጽ:	text
54	dss_r	Section Header: *Healthcare services for pregnant women Tajaajila Kunuunsa Fayyaa dubartoota Ulfaaf (R)* ለነፍሰ ጡር ሴቶች የጤና እንክብካቤ አገልግሎቶች<br>R. Is there a woman in the household who has been pregnant since mid-March?R. Dubartoonni yeroo giddu gala bitootessaa sana Ulfa ta'an ni jiruu?R. በቤት ውስጥ ከመጋቢት አጋማሽ ጀምሮ ነፍሰ ጡር የሆነች ሴት ይኖር ይሆን?	radio<br>CH00001 – Yes**Eeyyeen **አዎ<br>CH00002 – No**Lakki**አይ
55	dss_rinstruct<br>Show the field ONLY if:<br>[dss_r] = 'CH00001'	I would like to ask about the pregnancy health services received during this time.Ulfaa kanaaf gargaarsaa fayyaa fudhattee ilaalchisee gaafilee siin gaafadhaለዚህ እርግዝና የህክምና አገልግሎት አወሳሰድ መጠይቅ	descriptive
56	dss_r1<br>Show the field ONLY if:<br>[dss_r] = 'CH00001'	R1. Since mid-March, did you attend any pregnancy-related healthcare? R1. Giddu gala bitootessaatti,Tajaajila fayyaa da'umsa waliin wal qabate kamiyyuu haa tahu kan argatte ni jiraa?R1.ከመጋቢት አጋማሽ አንስቶ ከእርግዝና ጋር በተዛመደ የጤና እንክብካቤ ተገኝተዋል?	radio<br>CH00001 – Yes**Eeyyeen **አዎ<br>CH00002 – No**Lakki**አይ<br>98 – Don't know**Hin beeku**አላውቅም<br>99 – Refused to respond**Deebiif eeyyamamaa tahuu dhabuu**ለመመለስ ፈቃደኛ አልሆነም/ችም

57	dss_r2<br><br>Show the field ONLY if:<br>[dss_r1] = 'CH00001'	R2. What kind of healthcare during pregnancy was received since mid-March?<br>(PLEASE DO NOT READ, SELECT ALL THAT APPLY)R2. yeroo ulfaa giddu galeessa Bitootessaatti gosa tajaajila fayyaa kam fudhattee?<br>(MAALOO HIN DUUBISN, DEEBI FILATAMEE HUNDAA GUUTI)R2. ከመጋቢት አጋማሽ ጀምሮ በእርግዝና ወቅት ምን ዓይነት የጤና እንክብካቤ ተቀበለ ?<br>(እባክዎ አያነቡ ፣ ለሚመለከታቸው ሁሉ ይምረጡ)	checkbox<br>01 | dss_r2___01 | Routine antenatal visits to clinics/hospital**Beellama Tajaajila da'umsa duraatiif Kilinika/Hospitaalatti**ለመደበኛ የቅድመ ወሊድ ጉብኝት ወደ ክሊኒኮች / ሆስፒታል<br>02 | dss_r2___02 | Clinic visits for pregnancy-related complication or concern**Rakkoo Ulfa waliin wal qabate kilinikatti ilaalamuuf**ከእርግዝና ጋር በተዛመደ ክሊኒክ የሚደረግ ጉብኝት<br>03 | dss_r2___03 | Delivery at clinic/hospital**Da'umsa kilinikatti/hospitalitti**በክሊኒክ / ሆስፒታል መዉለድ<br>04 | dss_r2___04 | C-section**Da'umsa baqaqsanii baasaniif**በቀዶ ህክምና መውለድ<br>05 | dss_r2___05 | Clinic visit for any illness not related to the pregnancy** Dhukkuba biroo ulfa waliin wal hin qabanne kilinikatti ilaalamuuf**ከእርግዝና ጋር የማይዛመድ ለማንኛውም ህመም ክሊኒክ መጎብኘት<br>06 | dss_r2___06 | Did not get medications**Qoricha fudhatamuu hin argannee**መድኃኒቶችን አላገኘሁም<br>07 | dss_r2___07 | Routine postnatal visit at clinic/hospital**Beellama da'umsa boodaa kilinika/Hospitaalattii** መደበኛ የድህረ ወሊድ ጉብኝት በክሊኒክ / በሆስፒታል<br>09 | dss_r2___09 | Clinic visits for postnatal concern or complications**Rakkoo da'umsa boodaa waliin walqabate kilinikatti ilaalamuuf**ለድህረ ወሊድ ጭንቀት ወይም ውስብስቦች ክሊኒክ መጎብኘት<br>88 | dss_r2___88 | Other**Kan biroo **ሌላ ካለ ይግለጹ<br>98 | dss_r2___98 | Don't know**Hin beeku**አላውቅም<br>99 | dss_r2___99 | Refused to respond**Deebiif eeyyamamaa tahuu dhabuu**ለመመለስ ፈቃደኛ አልሆነም/ችም
58	dss_r2other<br><br>Show the field ONLY if:<br>[dss_r2(88)] = '1'	Other specify:Kan biraa Ibsii:ሌላ ካለ ይገለጽ:	text
59	dss_r3<br><br>Show the field ONLY if:<br>[dss_r] = 'CH00001'	R3. Since mid-March, was there a time you needed medical care during the pregnancy but did not receive it?R3. Yeroo giddu gala Bitootessaatti tajaajila yeroo ulfaa barbaaddee turee garuu hin fudhatiin hafte ni jiraa?R3. ከመጋቢት አጋማሽ ጀምሮ በእርግዝና ወቅት የሕክምና እንክብካቤ የሚፈልጉበት ጊዜ አላገኙም?	radio<br>CH00001 | Yes**Eeyyeen **አዎ<br>CH00002 | No**Lakki**አይ<br>98 | Don't know**Hin beeku**አላውቅም<br>99 | Refused to respond**Deebiif eeyyamamaa tahuu dhabuu**ለመመለስ ፈቃደኛ አልሆነም/ችም

60	dss_r3a<br>Show the field ONLY if:<br>[dss_r3]='CH00001'	R3a. During this time, how many medical care or clinical visits were missed?R3a. Yeroo kanatti,tajaajila fayyaa yookiin beellama kilinikaarraa qabdu meeqatu si jala darbe?R3a. በዚህ ወቅት ስንት<br>የሕክምና እንክብካቤ ወይም ክሊኒካዊ ጉብኝቶች አምልጠዋል?	text
61	dss_r4<br>Show the field ONLY if:<br>[dss_r3] = 'CH00001'	R4. What kind of healthcare was needed but not received since mid-March<br>(PLEASE DO NOT READ, SELECT ALL THAT APPLY)R4. Yeroo giddu gala Botootessaatti Gosa tajaajila fayyaa kamtu barbaadamee garuu kan hin fudhatamne ?<br>(MAALOO HIN DUUBISN, DEEBI FILATAMEE HUNDAA GUUTI)R4. ምን ዓይነት የጤና እንክብካቤ ያስፈልግ ነበር ግን ከመጋቢት አጋማሽ ጀምሮ አልተቀበለም ? (እባክዎ አያነቡ ፣ ለሚመለከታቸው ሁሉ ይምረጡ)	checkbox<br>01 | dss_r4___01 | Routine antenatal visits to clinics/hospital**Beellama Tajaajila da'umsa duraatiif Kilinika/Hospitaalatti**ለመደበኛ የቅድመ ወሊድ ጉብኝት ወደ ክሊኒኮች / ሆስፒታል<br>02 | dss_r4___02 | Clinic visits for pregnancy-related complication or concern**Rakkoo Ulfa waliin wal qabate kilinikatti ilaalamuuf**ከእርግዝና ጋር በተዛመደ ክሊኒክ የሚደረግ ጉብኝት<br>03 | dss_r4___03 | Delivery at clinic/hospital**Da'umsa kilinikatti/hospitalitti**በክሊኒክ / ሆስፒታል መዉለድ<br>04 | dss_r4___04 | C-section**Da'umsa baqaqsanii baasaniif**በቀዶ ህክምና መውለድ<br>05 | dss_r4___05 | Clinic visit for any illness not related to the pregnancy** Dhukkuba biroo ulfa waliin wal hin qabanne kilinikatti ilaalamuuf**ከእርግዝና ጋር የማይዛመድ ለማንኛውም ህመም ክሊኒክ መጎብኘት<br>06 | dss_r4___06 | Did not get medications**Qoricha fudhatamuu hin argannee**መድኃኒቶችን አላገኘሁም<br>07 | dss_r4___07 | Routine postnatal visit at clinic/hospital**Beellama da'umsa boodaa kilinika/Hospitaalattii** መደበኛ የድህረ ወሊድ ጉብኝት በክሊኒክ / በሆስፒታል<br>09 | dss_r4___09 | Clinic visits for postnatal concern or complications**Rakkoo da'umsa boodaa waliin walqabate kilinikatti ilaalamuuf**ለድህረ ወሊድ ጭንቀት ወይም ውስብስቦች ክሊኒክ መጎብኘት<br>88 | dss_r4___88 | Other**Kan biroo **ሌላ ካለ ይግለጹ<br>98 | dss_r4___98 | Don't know**Hin beeku**አላውቅም<br>99 | dss_r4___99 | Refused to respond**Deebiif eeyyamamaa tahuu dhabuu**ለመመለስ ፈቃደኛ አልሆነም/ችም
62	dss_r4other<br>Show the field ONLY if:<br>[dss_r4(88)] = '1'	Other specify:Kan biraa Ibsii:ሌላ ካለ ይገለጽ:	text

63	dss_r5<br>Show the field ONLY if:<br>[dss_r3] = 'CH00001'	R5. Why was this care not received?<br>(PLEASE DO NOT READ, SELECT ALL THAT APPLY)R5. Maaliif tajaajila kana hin fudhanne?<br>(MAALOO HIN DUUBISN, DEEBI FILATAMEE HUNDAA GUUTI)R5. ይህ እንክብካቤ ለምን አልተቀበለም?<br>(እባክዎ አያነቡ ፣ ለሚመለከታቸው ሁሉ ይምረጡ)	checkbox<br>01 | dss_r5___01 | Clinic closed**kiliniki cufaa ture**ክሊኒክ ተዘግቷል<br>02 | dss_r5___02 | Out of vaccines or medications**Talaallii yookiin Qorichaan ala**ከክትባቶች ወይም መድሃኒቶች ዉጭ<br>03 | dss_r5___03 | Did not get transportation**Geejjiba hin arganne**መጓጓዣ አላገኘም<br>04 | dss_r5___04 | Lockdown**Dhorkinsa**ክልከላ<br>05 | dss_r5___05 | Scared to go**Deemuu Sodaachuu**ለመሄድ ፈርቷል<br>88 | dss_r5___88 | Other**Kan biroo **ሌላ ካለ ይግለጹ<br>98 | dss_r5___98 | Don't know**Hin beeku**አላውቅም<br>99 | dss_r5___99 | Refused to respond**Deebiif eeyyamamaa tahuu dhabuu**ለመመለስ ፈቃደኛ አልሆነም/ችም
64	dss_r5other<br>Show the field ONLY if:<br>[dss_r5(88)] = '1'	Other specify:Kan biraa Ibsii:ሌላ ካለ ይገለጽ:	text
65	dss_endtime	Section Header: *THE SURVEY HAS COMPLETED. INTERVIEWER SAY: We have completed the survey. Thank you very much for your participation, it contributes a lot to our study. We do appreciate your participation.Daataan funaaname Xumuramee jira.Namni gaafatu kan jedhu:Daataa funaannu xumurree jirra. Hirmaannaakeef baay'ee si galateeffanna Hirmaannaakeef baay'ee si dinqisiifannaጥናቱን አጠናቅቀናል ፡ ስለ ተሳትፎዎ በጣም አመሰግናለሁ ፣ ለጥናታችን ትልቅ አስተዋጽኦ አለው ፡ ተሳትፎዎን እናደንቃለን ፡*<br>Interview End Time:Sa'aati gaafin ittii xumuramee: መተይቁ ያለቀበት ሰአት:	text (time)
66	harmonized_covid19_impact_questions_for_champs_dss_complete	Section Header: *Form Status*<br>Complete?	dropdown<br>0 | Incomplete<br>1 | Unverified<br>2 | Complete
