# Supplementary material for: COVID-19 impact data for the CHAMPS HDSS network: Data from Harar and Kersa, Ethiopia
Source: Data Brief. 2023 Aug 21;50:109508. doi: 10.1016/j.dib.2023.109508 (PMC10471914; doi:10.1016/j.dib.2023.109508)
Supplement: Supplementary file 2 [file mmc2.docx]

**Article information**

**Article title**

COVID-19 Impact Data for the CHAMPS HDSS Network: Data from Harar and Kersa, Ethiopia

**Authors**

*Jonathan A. Muir1†*, Merga Dheresa2†, Zachary J. Madewell3, Tamirat Getachew2, Gamachis Daraje2, Gezahegn Mengesha2, Cynthia G. Whitney1, Nega Assefa2‡, and Solveig A. Cunningham1‡*

*†Co-first authors*

*‡Co-senior authors*

**Corresponding author*

**Affiliations**

1. Emory University, Atlanta, GA, United States
2. Haramaya University, Harar, Ethiopia
3. Global Health Center, Centers for Disease Control and Prevention, Atlanta, GA, United States

**Corresponding author’s email address and Twitter handle**

Jonathan A. Muir, Emory University, Atlanta, GA, United States, [jamuir@emory.edu](mailto:jamuir@emory.edu)

**Keywords**

SARS-CoV-2; East Africa; Resilience; Vulnerability

**Abstract**

Data were collected as part of the Child Health and Mortality Prevention Surveillance (CHAMPS) network to learn about the effects of COVID-19 lockdowns on child health and access to care. Data were collected between August and September 2021 through a Health and Demographic Surveillance System (HDSS) operating in Eastern Ethiopia using a survey instrument focused on knowledge about COVID-19 and changes in food availability and healthcare services during the COVID-19 related lockdown. The data are representative of two communities in Eastern Ethiopia, one rural (Kersa) and one urban (Harar), and consist of a random sample of 880 households.

**Specifications table**

| **Subject** | Public Health and Health Policy |
| --- | --- |
| **Specific subject area** | Household economic and social circumstances during the COVID-19 pandemic. |
| **Type of data** | Table |
| **How the data were acquired** | Data were acquired using the survey instrument “Harmonized COVID-19 Impact Questions for the CHAMPS HDSS Network.” A standardized version of this instrument and a corresponding data dictionary are accessible through the CHAMPS Population Surveillance data repository hosted by UNC Dataverse (https://doi.org/10.15139/S3/CZO1IX).[1] Data collection was carried out using the RedCAP platform for electronic data collection.[2] The data collection procedure adhered to a detailed protocol developed by the coauthors and was approved by an Institutional Health Research Ethics Review Committee (IHRERC); approval reference number Ref.No.IHRERC/127/2021. A copy of the protocol is included as supplementary material. |
| **Data format** | Raw |
| **Description of data collection** | The data were collected from two areas in Eastern Ethiopia. One was a predominantly rural area in the district of Kersa and the other was an urban area in the Harari People’s National Regional State.[3,4] The rural area encompasses 24 kebeles (local government subdivisions representing neighborhoods or wards), spans an area of 353 km^2^, and comprises a population of 135,754 individuals residing in 25,653 households. The urban area comprises 12 kebeles, covers an area of 25.4 km^2^, and has a population of 55,773 individuals residing in 14,768 households. These populations have been followed through a HDSS since 2012, with demographic and health-related information gathered during regular data collection rounds that historically occurred two to three times per calendar year. The regularly collected HDSS data are separate and distinct from the data described herein that are specific to information on COVID-19 impacts for households.  Households were selected from the HDSS using simple random sampling with a sample size set to 440 each from the rural (Kersa) and urban (Harar) catchment areas (total sample size of 880 households). The sample size was specified to detect prevalence of changes in accessing healthcare. *A priori* specifications were 50% of the population experiencing changes, 95% CI, precision of 0.05 and non-response adjustment of 10%. |
| **Data source location** | - Institution: Haramaya University and Emory University - City/Town/Region: Eastern Ethiopia - Country: Ethiopia |
| **Data accessibility** | Repository name: CHAMPS Population Surveillance Dataverse  Data identification number: https://doi.org/10.15139/S3/CZO1IX  Direct URL to data:  https://dataverse.unc.edu/dataset.xhtml?persistentId=doi:10.15139/S3/CZO1IX  Instructions for accessing these data: A deidentified version of the data are published and publicly available through the CHAMPS Population Surveillance Dataverse. |

**Value of the data**

- These data offer insights into household knowledge and awareness of COVID-19 as well as experiences of economic and social shocks associated with the pandemic and corresponding lockdowns, including impacts on child and maternal health and access to healthcare in a resource-limited setting, and thereby contribute to a better understanding of challenges faced by individuals and households during the pandemic period.
- Through offering insights into impacts of the COVID-19 pandemic and associated lockdowns on child and maternal health in a resource-limited setting, these data can inform future research and policy interventions developed by public health researchers and policy makers.
- These COVID-19 related data could be analyzed independently, in combination with demographic and or socioeconomic data from the Ethiopia HDSS where the data were collected, or in a pooled analysis with data from other CHAMPS HDSS sites. With additional data harmonization, these data could be leveraged for cross-site investigations that included data beyond the CHAMPS Network and thereby extend their utility and relevance.

**Objective**

The data described herein were gathered as part of a global health collaboration within the Child Health and Mortality Prevention Surveillance (CHAMPS) to understand implications of COVID-19 lockdowns and related social distancing policies for child health and mortality in resource-limited settings as well as how these lockdowns and related policies may have contributed to broader household economic and social shocks.[5-10] Specifically, they were gathered to enhance understanding of the impacts of the pandemic and associated lockdown measures on livelihoods, food availability, and healthcare services among populations residing within the geographic catchment areas of Harar and Kersa, Ethiopia that are affiliated with a network of HDSS sites within the CHAMPS network.[4]

**Data description**

The data, data dictionary, and survey instrument are available through a publicly accessible data repository hosted by Dataverse that is titled "CHAMPS Population Surveillance Dataverse.”

De-identified data from a random sample of 880 households are available for download through the repository are located in the file titled “Impact of Covid 19 From Harar and Kersa HDSS,” which was originally formatted as a comma-separated value (csv) table. Data included in the file are organized into distinct sections, corresponding to the modules of the survey instrument:

- Respondent and household information
  - Example variables: respondent age, sex, residence, marital status, educational level, occupation, and family size
- Knowledge of COVID-19
  - Example variables: awareness of COVID-19 preventive measures (hygiene, mask wearing, staying home, and social distancing) and governmental actions related to COVID-19 transmission (movement restriction, public awareness, isolation, and disinfection of public places)
- Food security
  - Example variables: food availability, food delivery services, food affordability, and fears associated with COVID-19 that inhibited purchasing food
- COVID-19 related economic shocks and coping
  - Example variables: household member job loss, business closure, and agricultural disruptions as well as household observation of price increases for food, agricultural inputs, and/or business inputs.
- Healthcare services for children and pregnant women
  - Example variables: routine ANC, PNC & delivery services; routine vaccination; malaria treatment; HIV treatment; chronic illness services; service for malnutrition; and COVID-19 testing

Variable names adhere to the naming convention outlined in the data dictionary/codebook (see supplementary material). For survey questions that asked respondents to indicate multiple options or “select all that apply”, responses were disaggregated into individual variables in the raw data file. The variable labels associated with these variables are designed to facilitate clear identification of the information they represent.

The data dictionary is available for download as a PDF file titled “Codebook-Data Dictionary.” The data dictionary is a 15-page document that organizes information about each of the variables included in the data into 4 columns (number, variable/field name, field label, and field attributes. It is a representation of the information contained in the electronic version of the survey that was implemented using tablets in the field. The field label provides a description of the information represented by a given variable and includes the English as well as the Amharic and Afaan Oromo terms. The field attribute column provides information about the type of variable that was collected as well as any variable values and value labels, which are provided in both English as well as translated Amharic and Afaan Oromo terms.

The survey instrument is titled “CHAMPS-COVID19-instrument” and includes the original survey instrument formatted for paper-based data collection. This survey instrument was adapted into an electronic format that included both English and translated Amharic and Afaan Oromo terms, which is presented in the data dictionary.

**Experimental design, materials and methods**

Data were collected in Eastern Ethiopia from a predominantly rural area in the district of Kersa and an urban area in the Harari People’s National Regional State.[3,4] The rural area covers 353 km^2^, which is divided into 24 kebeles (neighborhoods), and is the home of 135,754 individuals residing in 25,653 households. The urban area is home to a population of 55,773 individuals residing in 14,768 households; it spans across 25.4 km^2^ and is partitioned into 12 kebeles. Regular surveillance of the population began in 2012 with the establishment of an HDSS to collect demographic and health-related information.

Simple random sampling was used to select a sample of the households residing within the HDSS catchment areas. A target sample size was set to 440 households from Kersa and another 440 households from Harar. These target sample sizes were achieved, resulting in a combined sample size of 880 households. The sample size was specified to detect prevalence of changes in accessing healthcare. A priori specifications were 50% of the population experiencing changes, 95% CI, precision of 0.05 and non-response adjustment of 10%.

The data collection instrument was developed using established survey design methods.[11] These methods included identification of key research questions and concepts; conducting a rapid review of pertinent literature; and identification of example survey questionnaires (e.g., the “High Frequency Mobile Phone Surveys of Households to Assess the Impacts of COVID-19” questionnaire that was developed by the World Bank as a standardized instrument for assessing social and economic impacts of the pandemic), which were used as preliminary source material from which a subset of survey questions were selected and revised.[12,13] A harmonized instrument was generated as a template for use across the CHAMPS network, which was revised slightly for implementation within local contexts. The finalized data collection instrument was organized into six sections: respondent information, knowledge regarding the spread of COVID-19; food availability; COVID-19 related shocks/coping; under-five child healthcare services; and healthcare services for pregnant women (see supplementary materials). The survey instrument included questions related to hardships encountered during the pandemic period, prompting respondents to reflect on their experiences since March 2020. Data collection was carried out using the RedCAP platform for electronic data collection, which included translations of the English version of the instrument into Amharic and Afaan Oromo.[2]

Data cleaning and validation followed standard operating procedures for the HDSS.[3,14] Data quality control included pretesting the survey instrument on a sub-sample of respondents who were not eligible for the study. Findings and experiences from the pretest were utilized in revising the research data collection tools. Data collectors and supervisors were provided training concerning the objective of the study, confidentiality of information, and techniques of data collection. Data concerns such as implausible values, data inconsistencies, and/or incomplete data were flagged for in-field correction by the data collectors. A random sample of questionnaires were selected for re-visits by field supervisors and the field coordinator to verify the recorded information. Implementation of the data collection module was approved by the Institutional Health Research Ethics Review Committee (IHRERC); approval reference number Ref.No.IHRERC/127/2021.

**Ethics statements**

This study was conducted according to the guidelines in the Declaration of Helsinki; all procedures involving research study participants, including digital data collection using tablets that were programmed with the corresponding survey instruments, were approved by the Institutional Health Research Ethics Review Committee (IHRERC), College of Health and Medical Sciences, Harar Campus, Ethiopia; approval reference number Ref.No.IHRERC/127/2021. Written informed consent was obtained for participants who were able to read and write. For participants who were unable to read or write, the informed consent statement was read and oral informed consent from the participant was obtained, recorded, and witnessed. These procedures for obtaining written or oral informed consent were approved by the Institutional Health Research Ethics Review Committee (IHRERC), College of Health and Medical Sciences, Harar Campus, Ethiopia; approval reference number Ref.No.IHRERC/127/2021.

**CRediT author statement**

J.M., Z.M., and M.D provided project administration. J.M., Z.M., S.C., N.A., M.D., and C.W. performed study conceptualization and methodology development. M.D., N.A., T.G., G.M., and G.D. performed data curation. J.M., Z.M., T.G., G.M., and G.D carried out data cleaning and validation. J.M. wrote the original draft. All authors reviewed and edited the manuscript. C.W., S.C., and N.A provided supervision for the study. C.W. acquired funding.

**Acknowledgments**

This work was supported by grant OPP1126780 from the Bill & Melinda Gates Foundation to Dr Cynthia Whitney. Under the grant conditions of the Foundation, a Creative Commons Attribution 4.0 Generic License has already been assigned to the Author Accepted Manuscript version that might arise from this submission. **Disclaimer**: The findings and conclusions in this report are those of the author(s) and do not necessarily represent the official position of the US Centers for Disease Control and Prevention.

**Declaration of interests**

The authors declare that they have no known competing financial interests or personal relationships that could have appeared to influence the work reported in this paper.

**References**

[1] [dataset] M. Dheresa, J.A. Muir, Z.J. Madewell, T. Getachew, G. Daraje, G. Mengesha, C.G. Whitney, N. Assega, S.A. Cunningham, COVID-19 Impact Data for the CHAMPS HDSS Network: Data from Harar and Kersa, Ethiopia, UNC Dataverse, VV1, 2023. <https://doi.org/doi:10.15139/S3/CZO1IX>.

[2] P.A. Harris, R. Taylor, B.L. Minor, V. Elliott, M. Fernandez, L. O'Neal, L. McLeod, G. Delacqua, F. Delacqua, J. Kirby, The RedCAP Consortium: Building an International Community of Software Platform Partners, Journal of Biomedical Informatics 95 (2019) 103208.

[3] N. Assefa, L. Oljira, N. Baraki, M. Demena, D. Zelalem, W. Ashenafi, M. Dedefo, HDSS profile: the Kersa Health and Demographic Surveillance System, International Journal of Epidemiology 45(1) (2016) 94-101.

[4] S.A. Cunningham, N.I. Shaikh, A. Nhacolo, P.L. Raghunathan, K. Kotloff, A.M. Naser, M.M. Mengesha, S.A. Adedini, T. Misore, U.U. Onuwchekwa, Health and Demographic Surveillance Systems within the Child Health and Mortality Prevention Surveillance Network, Clinical Infectious Diseases 69(Supplement_4) (2019) S274-S279.

[5] J. Farley, A. Osterman, S.E. Hawes, K. Martin, S.J. Morrison, K.K. Holmes, Global health programs and partnerships: evidence of mutual benefit and equity, Rowman & Littlefield2016.

[6] J.A. Muir, J. Farley, A. Osterman, S.E. Hawes, K. Martin, J. Stephen Morrison, K.K. Holmes, Global health partnerships: are they working?, American Association for the Advancement of Science, 2016, pp. 334ed4-334ed4.

[7] J.A. Muir, M. Deresa, Z.J. Madewell, T. Getachew, G. Mangesha, C.G. Whitney, N. Assefa, S.A. Cunningham, Household Hardships during the COVID-19 Pandemic: Examining Household Vulnerability and Responses to Pandemic Related Shocks in Eastern Ethiopia, medRxiv (2023) 2023.02. 01.23285322.

[8] J.A. Muir, M. Deresa, Z.J. Madewell, T. Getachew, G. Mengesha, C.G. Whitney, N. Assefa, S.A. Cunningham, Food Insecurity amid COVID-19 Lockdowns: Assessing Sociodemographic Indicators of Vulnerability in Harar and Kersa, Ethiopia, medRxiv (2023) 2023.01. 31.23284545.

[9] A. Nhacolo, Z.J. Madewell, J.A. Muir, C. Sacoor, E.G. Xerinda, T. Matsena, Q. Bassat, C.G. Whitney, I. Mandomando, S.A. Cunningham, Knowledge of COVID-19 Symptoms, Transmission, and Prevention: Evidence from Health and Demographic Surveillance in Southern Mozambique, medRxiv (2023) 2023.03. 31.23288026.

[10] J.A. Muir, Societal Shocks as Social Determinants of Health, The Ohio State University2021.

[11] E. Ruel, W.E. Wagner III, B.J. Gillespie, The practice of survey research: Theory and applications, Sage Publications2015.

[12] World Bank Group, High Frequency Mobile Phone Surveys of Households to Assess the Impacts of COVID-19 (Vol. 4) : Questionnaire Template (English), Washington, D.C., 2020.

[13] A. Palacios-Lopez, C. Noumedem Temgoua, D. Kokas, E.A. Talbert, G.O. Siwatu, G. Ponzini, J. Illukor, L. Phadera, N. Yoshida, P.R. Wollburg, High Frequency Mobile Phone Surveys of Households to Assess the Impacts of COVID-19 (Vol. 5): Overview of the Questionnaire Template, World Bank Group, Washington, D.C., 2020.

[14] S.A. Cunningham, J.A. Muir, Data Cleaning, in: A.L. Nichols, J.E. Edlund (Eds.), The Cambridge Handbook of Research Methods and Statistics for the Social and Behavioral Sciences: Volume 1: Building a Program of Research (Cambridge Handbooks in Psychology, pp. 443-467), Cambridge University Press, Cambridge, 2023. <https://doi.org/doi:10.1017/9781009010054.022>.
